# Gilteritinib-induced pyoderma gangrenosum in a patient with acute myeloid leukemia: A case report

**DOI:** 10.1016/j.jdcr.2024.12.034

**Published:** 2025-01-24

**Authors:** Jonathan Sisley, Mallory deCampos-Stairiker, Sarah L. Becker, Shannon K. Throckmorton, Ronan Swords, Alex G. Ortega-Loayza

**Affiliations:** a Oregon Health & Science University School of Medicine, Portland, Oregon; b Oregon Health & Science University Department of Dermatology, Portland, Oregon; c Oregon Health & Science University Department of Hematology/Medical Oncology, Portland, Oregon

**Keywords:** cutaneous toxicity, drug-induced, neutrophilic dermatosis, pyoderma gangrenosum

## Introduction

Pyoderma gangrenosum (PG) is a rare, ulcerative neutrophilic dermatosis that often arises idiopathically but can manifest in patients with inflammatory bowel disease, rheumatologic arthritis, or hematological disorders, with the latter representing a marginal population of PG patients.^1^ The pathogenesis of PG is not completely understood, but drug-induced cases offer a unique model. Numerous prior studies have reported FMS-like tyrosine kinase 3 (FLT3) mutations in association with neutrophilic dermatoses (NDs).^1^^,^^2^ The FLT3 gene is a component of the tyrosine-kinase pathway, producing FLT3, which is responsible for promoting hematopoietic cell differentiation. FLT3 mutations represent one of the common gene mutations in acute myeloid leukemia (AML), and they can be targeted with an FLT3 inhibitor such as gilteritinib.^3^ Gilteritinib has been identified as a trigger of other NDs, including Sweet syndrome, but previous cases of gilteritinib-induced PG have not been reported.^1^^,^^2^

## Case report

**Fig 1:**
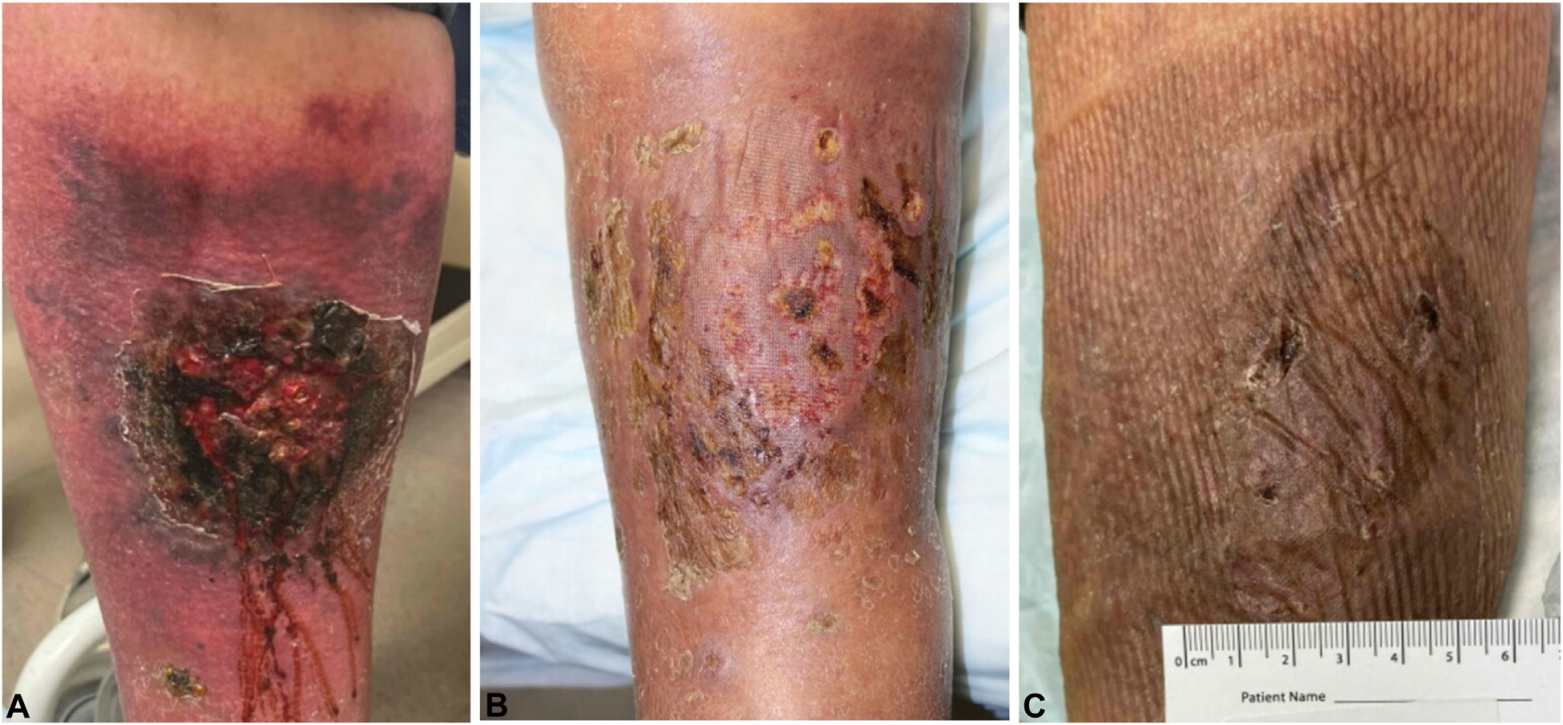
(*Left* to *right*): Ulcer healing over 2 months following withdrawal of gilteritinib and treatment with topical steroid. (a) Ulcer at presentation (*left*). (b) Ulcer 1 month later (*center*). (c) Ulcer site 2 months later (*right*).

**Fig 2:**
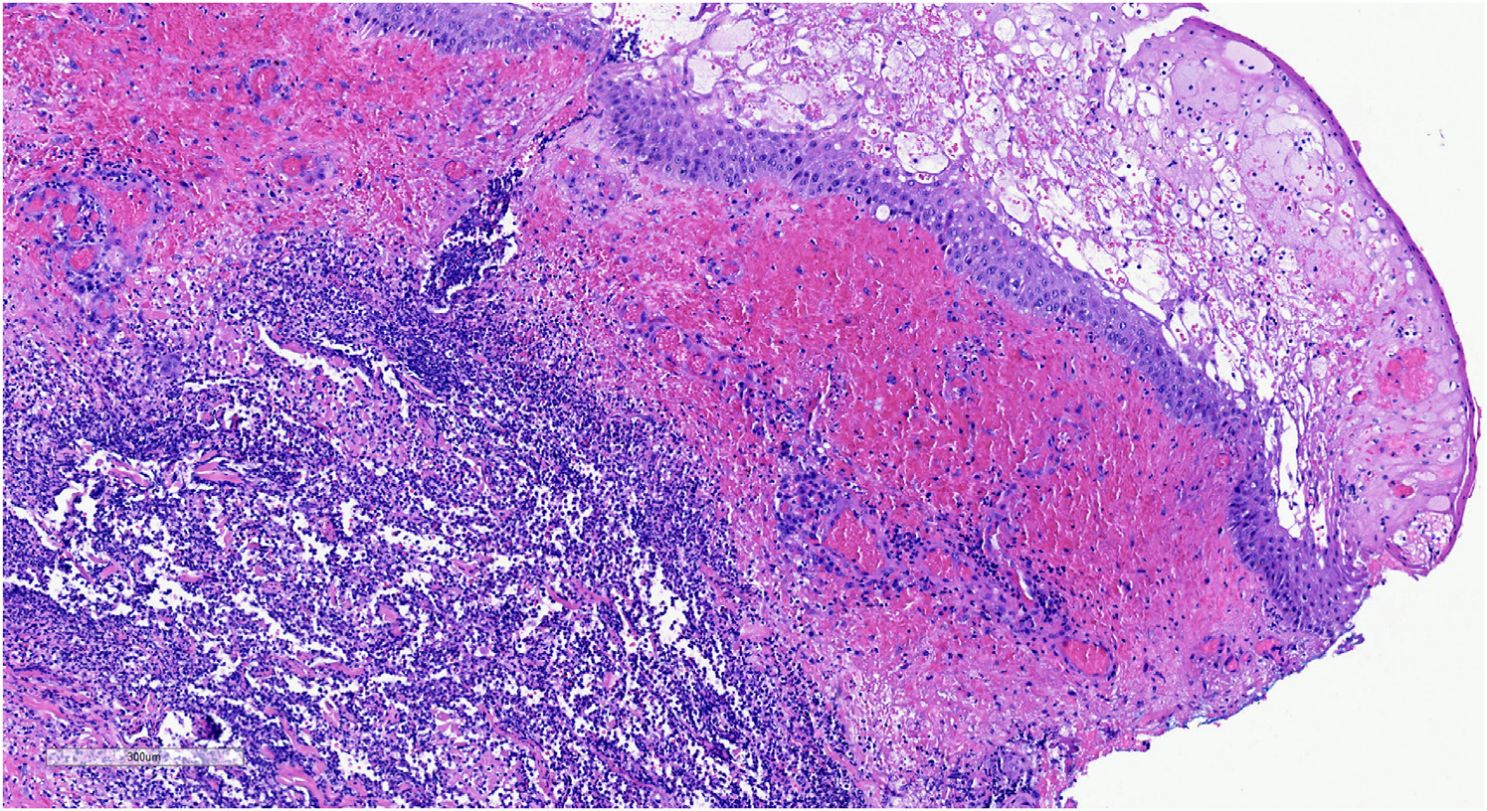
Histology of skin biopsy showing perivascular and interstitial lymphohistiocytic mixed infiltrate extending from the superficial to deep dermis and into the upper subcutis, with numerous neutrophils, plasma cells, and erythrocytes. Findings also include karyorrhexis with necrosis and fibrin exudation surrounding blood vessels.

We present a case of an 83-year-old male with pancytopenia undergoing treatment with gilteritinib for relapsed AML who acutely developed painful skin ulcerations on both lower extremities 2 weeks after initiating gilteritinib. Physical exam revealed an 8 cm × 10 cm necrotic ulcerated plaque on the right medial lower extremity (Fig 1, *A*). Similar ulcerations were noted on the left medial and lateral ankle and left second and fifth digits. Upon clinical evaluation, the differential included angio-invasive infection, leukemia cutis, and drug-induced PG. Skin biopsy revealed diffuse neutrophilic infiltration in the dermis and subcutaneous tissues with negative microbiological cultures (Fig 2). His clinical presentation, together with a PARACELSUS score >10, supported PG as the most likely diagnosis.^4^

Given the temporal relationship between gilteritinib initiation and symptom onset, drug-induced PG was suspected. Gilteritinib was held and voriconazole was initiated, given the concern for possible deep angio-invasive fungal infection. His ulcers rapidly improved within 2 weeks, and he achieved complete resolution in 3 months with addition of clobetasol 0.05% ointment, which further supported gilteritinib as the inciting agent. Ultimately, the patient passed due to relapsed AML 8 months after resolution of his ulcers.

## Discussion

In this case of suspected drug-induced PG in a pancytopenic patient with AML, the complex interaction between the underlying hematologic malignancy and drug therapy provides unique insights into the pathogenesis of PG. The pathogenesis of NDs, such as PG, in a pancytopenic patient is unknown but is likely multifactorial. Bone marrow failure in AML will occur in large part due to blast replacement of marrow, which results in pancytopenia.^5^ NDs have been associated with numerous inflammatory and myelodysplastic conditions and involve aberrant cellular signaling pathways.^3^ FLT3 inhibitors are a type of tyrosine kinase inhibitor (TKI) and target cell signaling, thereby promoting cell survival, proliferation, and differentiation through several pathways including phosphoinositide 3-kinase, signal transducer and activator of transcription 5, and rat sarcoma. Imatinib, a FLT3 inhibitor that targets a different enzyme binding site than gilteritinib, has also been associated with NDs, which suggests a possible common role of tyrosine kinase modulation in ND pathogenesis.^6^

**Fig 3:**
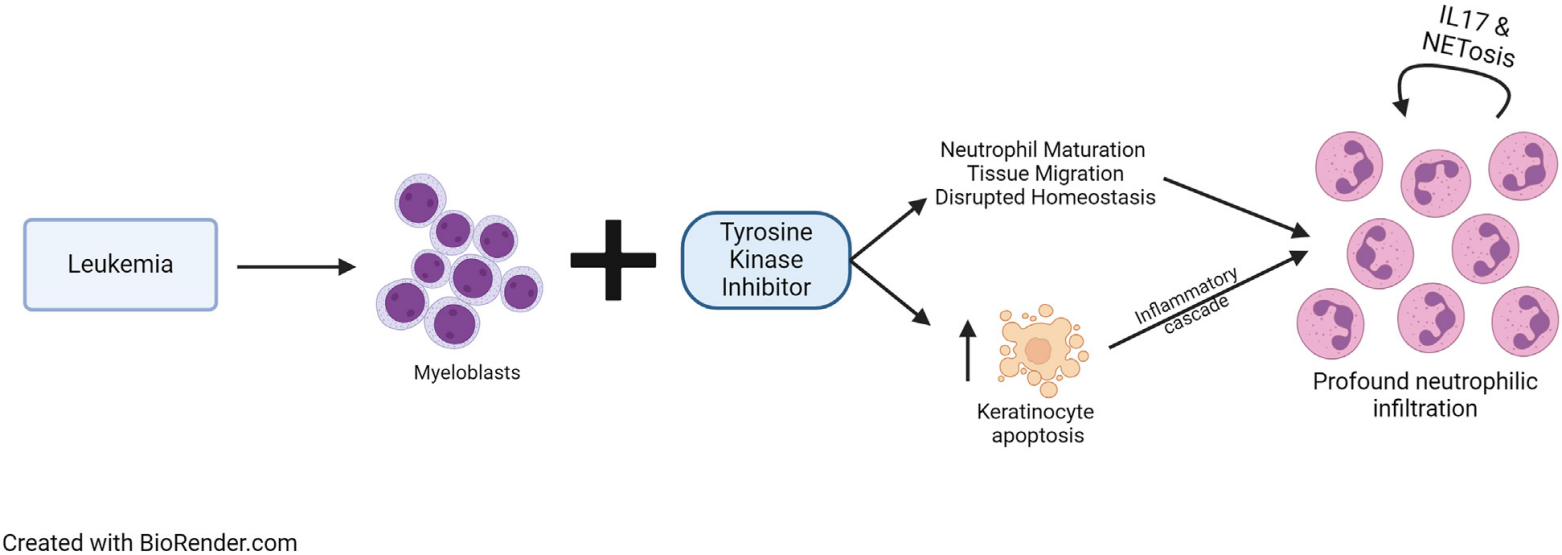
Proposed role of tyrosine kinase inhibitors in increasing susceptibility to neutrophilic dermatoses such as pyoderma gangrenosum. *IL*, Interleukin.

One mechanism of TKI-induced NDs may be disruption of neutrophil homeostasis through abrupt promotion of myelopoiesis.^6^ The exact mechanism of aberrant neutrophil migration to the skin in PG is unclear, but hypotheses include TKIs promotion of neutrophil maturation and cell migration. Therefore, neutrophilic migration to the skin in NDs in the setting of leukemia may be a sequela of clonal proliferation of leukemic cells with accumulation of clonal neutrophils in the dermis (Fig 3). This is further supported by observations of clonicity between malignant cells and dermal infiltrate, which are seen in other NDs, including Sweet syndrome.^7^ Thus, despite low circulating peripheral neutrophil counts in the setting of AML-induced pancytopenia, neutrophils that are present and migrating to tissue may be functionally abnormal.

Previous cases of multikinase inhibitor-induced neutrophilic dermatoses have been reported and may provide further insight into the pathogenesis of drug-induced PG.^6^ Multikinase inhibitors, such as sunitinib and pazopanib, also result in downstream activation of signal transducer and activator of transcription 3 (STAT3). Such drugs have been shown to induce keratinocyte apoptosis through increased levels of proapoptotic FasL and suppression of B-cell leukemia/lymphoma 2 protein (Bcl-2) and myeloid cell leukemia 1, as well as increased neutrophil recruitment and release of proinflammatory cytokines.^8^ FasL is a key regulator of the extrinsic pathway of apoptosis, initiating cell death upon binding to its receptor, Fas, on the cell surface. In contrast, Bcl-2 and myeloid cell leukemia 1 are members of the Bcl-2 protein family, which play critical roles in inhibiting apoptosis by maintaining mitochondrial integrity and preventing the release of cytochrome c, thus preserving cell survival. Additionally, STAT3 increases interleukin (IL)-17, a cytokine associated with NETosis. NETosis is a form of cell death exhibited by neutrophils that involves the creation of extracellular traps composed of chromatin and neutrophil granules that sequester and destroy pathogens. STAT3-associated increases in IL-17 contribute to excessive NETosis and are implicated in the pathogenesis of PG.^9^ Taken together, the addition of a TKI in the setting of underlying tissue-specific neutrophilia secondary to leukemia may result in increased susceptibility to keratinocyte apoptosis, triggering a localized inflammatory cascade with profound neutrophilic infiltration perpetuated by IL-17-induced NETosis.

In summary, this case highlights the potential role of tyrosine kinase inhibitors in triggering NDs, such as PG, through complex signaling pathways involving keratinocyte apoptosis and excessive neutrophil activation. The interplay between drug-induced signaling changes and pre-existing hematologic conditions underscores the need for heightened awareness and further exploration of these mechanisms. Continued research is essential to better understand the molecular drivers of drug-induced NDs, which might also improve our understanding of the pathophysiology of PG.

## Conflicts of interest

Dr Ortega-Loayza is President of the Pacific Dermatologic Association, a member of the Honorary Editorial Board of the American Journal of Clinical Dermatology, and Associate Editor of Dermatology (Karger). He has served as a consultant for Genentech, Guidepoint, Corvus Pharmaceuticals, Castle Biosciences, Clarivate, InflaRx GmbH, TFS HealthScience, and Otsuka. He is on the advisory board for Bristol Myers Squibb, Boehringer Ingelheim, Janssen, Sanofi, and Biotech and has received research grants from AbbVie, Lilly, Janssen, Pfizer, Incyte, Boehringer Ingelheim, and InflaRx GmbH. He is also supported by NIH NIAMS R01 AR083110. The other authors have no conflicts of interest to disclose.
